# Placental transmogrification of the lung: two case reports and a literature review

**DOI:** 10.3389/fmed.2025.1620403

**Published:** 2025-09-02

**Authors:** Hong-yuan Zhou, Hong Li, Yu-jie Lu, Heng-ping Wu, Yong-zhen Li

**Affiliations:** ^1^Department of Pathology, The First People’s Hospital of Zigong, Zigong, China; ^2^Department of Radiology, The First People’s Hospital of Zigong, Zigong, China

**Keywords:** placental transmogrification of the lung, giant bullae, papillary structures, mesenchymal tumor, CD10

## Abstract

**Background:**

Placental transmogrification of the lung (PTL) is an uncommon benign lung lesion characterized by the presence of immature mesenchymal clear cells and frequent cystic changes.

**Case presentation:**

We report two cases. The first case was a 51-year-old woman who presented with chest pain and a history of pulmonary nodule detected during examination for over 1 month. The second case was a 58-year-old man with a smoking history who presented with progressive dyspnea and cough for over 1 month, and was found to have an asymptomatic pulmonary lesion. Computed tomography revealed localized cystic lesions in the first case and bullae in the second. Histological examination showed papillary structures resembling placental villi, lined by cuboidal epithelium without atypia, with hyalinized stromal cores containing capillaries and lymphoplasmacytic infiltration.

**Conclusion:**

PTL exhibits placental-like architecture with CD10-positive stromal cells, suggesting it is a benign mesenchymal tumor rather than a form of emphysema. Pathological confirmation is essential, and surgical resection is the recommended treatment.

## 1 Introduction

Placental transmogrification of the lung (PTL), which is also known as placental-like bullous lesions of the lung, is a rare, benign condition that was first described by McChesney in 1978 (1). This condition was named PTL because its lesions morphologically resemble immature placental villi, without the biological or biochemical properties of a placenta (2). In imaging studies, most PTL cases are reported as exhibiting bullous emphysema changes, which ultimately lead to the occurrence of pneumothorax. Therefore, some scholars believe that PTL is a variant or secondary change of bullous emphysema (3–5); however, the pathogenesis of PTL remains unclear. This article reports two cases of PLT that were diagnosed between November 2023 and December 2024 and presents a comprehensive retrospective analysis of PLT, combining all relevant literature. The analysis included age of onset, sex, clinical manifestations, imaging features, histopathological characteristics, and treatment methods. On the basis of these findings, this study explored the pathogenesis of PLT to provide clinicians and pathologists with more information about its diagnosis and treatment. This report presents a histological, immunohistochemical, ultrastructural and molecular study of these peculiar cells, together with a literature review.

## 2 Case presentation

### 2.1 Case 1

**Figure 1:**
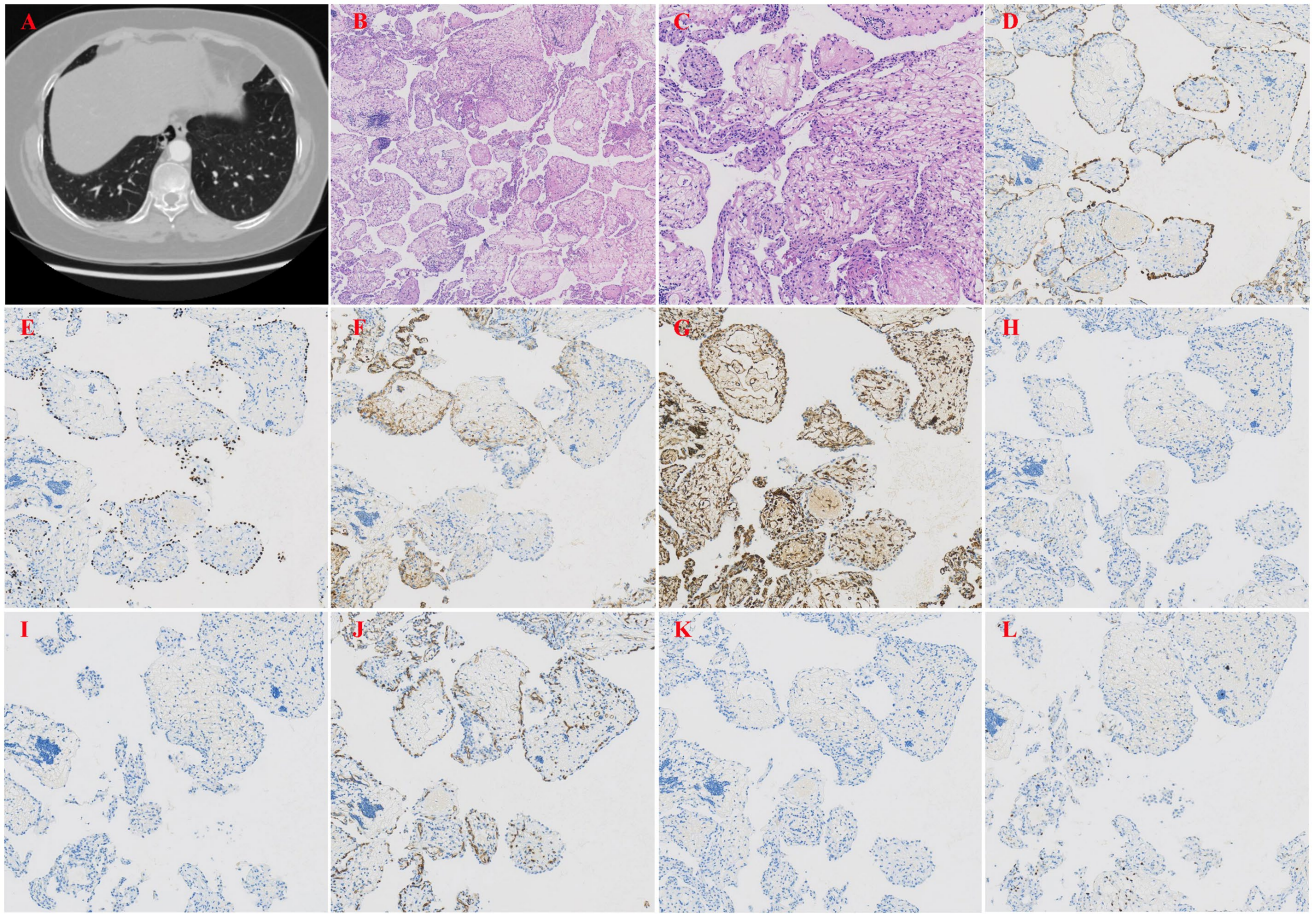
**(A)** Chest CT revealed a patchy area of increased density in the medial basal segment of the right lower lobe. **(B)** Papillary structures resembling mature placental villi (H&E, x40). **(C)** The papillae were lined by a single layer of cuboidal epithelial cells without atypia (H&E, x200). **(D)** IHC staining showed that the epithelial cells were positive for pancytokeratin (P-CK), indicating epithelial origin (H&E, x100). **(E)** IHC staining showed that the epithelial cells were positive for thyroid transcription factor-1 (TTF-1), a marker of respiratory epithelial cells (H&E, x100). **(F)** IHC staining showed that the stromal cells were positive for CD10, a metalloproteinase involved in extracellular matrix remodeling (H&E, x100). **(G)** IHC staining showed that the stromal cells were positive for Vimentin, a marker of mesenchymal cells (H&E, x100). **(H)** IHC staining showed that both epithelial and stromal cells were negative for Desmin, excluding smooth muscle differentiation (H&E, x100). **(I)** IHC staining showed that the epithelial cells were positive for HMB45, a marker typically used to identify melanocytic or perivascular epithelioid cells (H&E, x100). **(J)** IHC staining showed that both epithelial and stromal cells were negative for CD34, excluding vascular endothelial origin (H&E, x100). **(K)** IHC staining showed that both epithelial and stromal cells were negative for HCG, helping to rule out gestational trophoblastic disease (H&E, x100). **(L)** Ki-67 staining showed low proliferative activity (approximately <5%), consistent with a benign lesion (H&E, x100).

The patient was a 51-year-old female who experienced chest pain 1 month prior and had no history of smoking. A chest CT scan (Figure 1A) revealed a patchy area of increased density in the medial basal segment of the right lower lobe, measuring approximately 1.6 × 0.9 cm at its largest cross-section, and this area contained a small cavity. The patient self-administered oral anti-inflammatory medication. A follow-up chest CT revealed a patchy area of increased density in the medial basal segment of the right lower lobe, with a small cavity, similar to the results of the previous scan. As the lesion was small, located deep in the lung, and associated with cystic changes, fine-needle aspiration was considered to have low diagnostic yield. Therefore, under thoracoscopy, wedge resection of the right lower lobe was performed. Gross examination revealed a piece of lung tissue with a gray–red, cystic-solid nodule that was located adjacent to the pleura. The nodule measured 1.7 cm × 1.5 cm × 0.8 cm. The cut surface of the nodule was gray–red, cystic-solid, and soft, with clear boundaries from the surrounding lung tissue. Pathological examination under low-power microscopy revealed endophytic papillary structures of varying sizes that resembled mature placental villi (Figure 1B). High-power microscopy revealed papillae lined with a single layer of cuboidal epithelial cells without atypia (Figure 1C); the papillary cores were composed of dense hyalinized stromal cells and abundant newly formed capillaries and exhibited varying degrees of lymphocyte infiltration. Immunohistochemistry (IHC) revealed that the surface cells were positive for P-CK (Figure 1D), CK7, TTF-1 (Figure 1E), NapsinA, and EMA expression, whereas the stromal cells were positive for CD10 (Figure 1F), Vimentin (Figure 1G) and CD117 (focal) expression. Both the surface and stromal cells were negative for CEA, Desmin (Figure 1H), S100, HMB45 (Figure 1I), CD68, CD34 (Figure 1J), SMA, and HCG (Figure 1K) expression, and Ki-67 staining revealed low proliferative activity (approximately <5%) (Figure 1L). The pathological diagnosis was PTL in the right lower lobe.

### 2.2 Case 2

**Figure 2:**
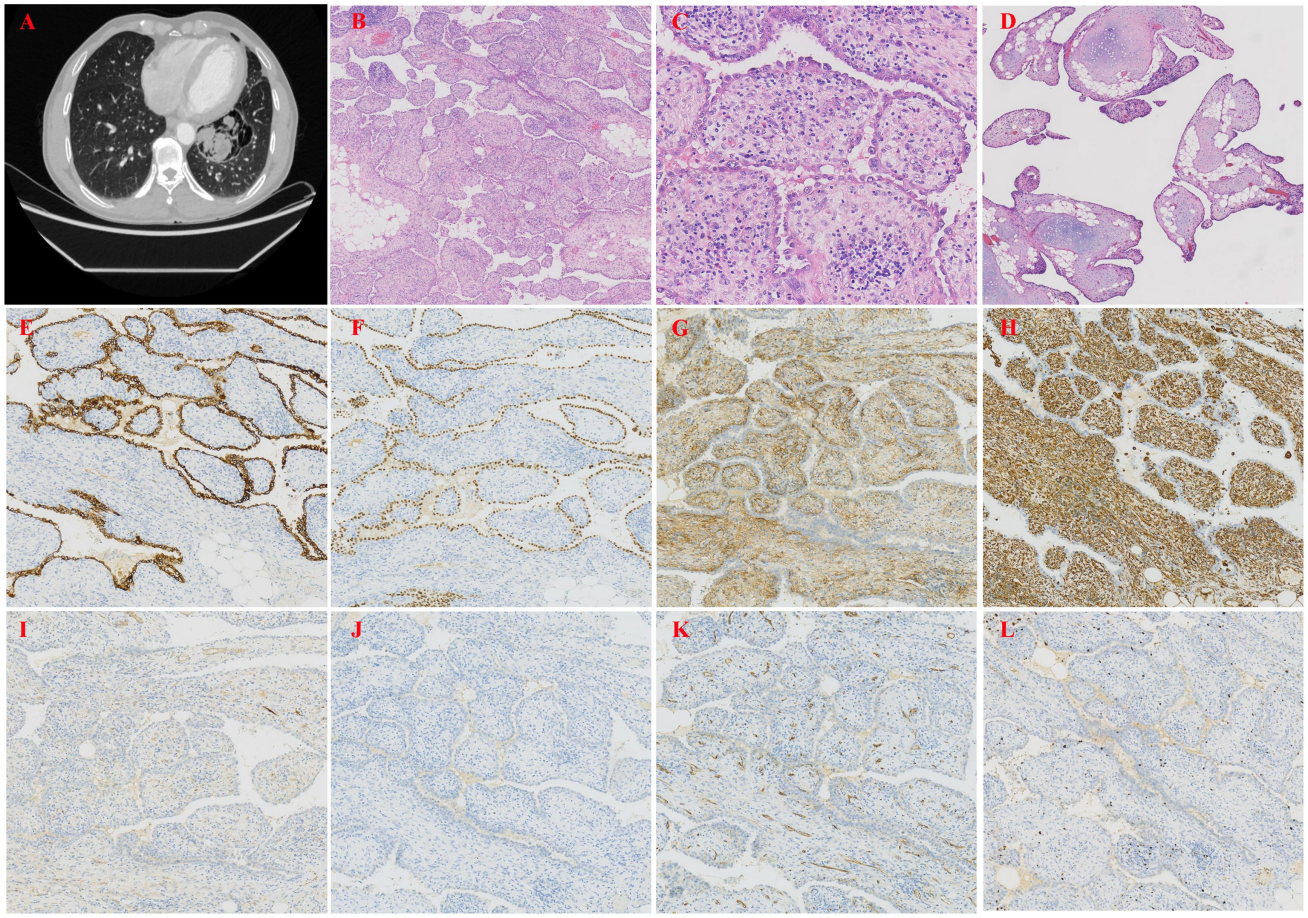
**(A)** Chest CT showed a giant bulla in the left upper lobe. **(B)** Papillary structures morphologically resembling mature placental villi (H&E, ×40). **(C)** The papillae were lined by a single layer of cuboidal epithelial cells without atypia (H&E, ×200). **(D)** Papillary structures with cartilage and lymphoid aggregates (H&E, ×40). **(E)** IHC staining showed that the epithelial cells were positive for P-CK, indicating epithelial origin (H&E, ×100). **(F)** IHC staining showed that the epithelial cells were positive for TTF-1, a marker of respiratory epithelial cells (H&E, ×100). **(G)** IHC staining showed that the stromal cells were positive for CD10, a metalloproteinase involved in extracellular matrix remodeling (H&E, ×100). **(H)** IHC staining showed that the stromal cells were positive for Vimentin, a marker of mesenchymal cells (H&E, ×100). **(I)** IHC staining showed that both epithelial and stromal cells were negative for Desmin, excluding smooth muscle differentiation (H&E, ×100). **(J)** IHC staining showed that the epithelial cells were positive for HMB45, a marker typically used to identify melanocytic or perivascular epithelioid cells (H&E, ×100). **(K)** IHC staining showed that both epithelial and stromal cells were negative for CD34, excluding vascular endothelial origin (H&E, ×100). **(L)** Ki-67 staining showed low proliferative activity in both epithelial and stromal cells (approximately 1%), consistent with a benign lesion (H&E, ×100).

A 58-year-old male was found to have a pulmonary lesion during a routine health examination more than 20 days prior, but the patient did not have symptoms such as dyspnea or cough. Additionally, the patient had a 30-year history of smoking. Five years ago, he underwent resection of a left pulmonary bulla due to spontaneous pneumothorax, with good postoperative recovery. This case was previously published as a case report by colleagues from the radiology department of our hospital in 2024, and the report focused on the imaging features of the disease (6). In that article, the imaging characteristics of this case were described in detail; these characteristics included features that were observed on chest X-ray and CT scans, such as giant bullae in the left upper lobe, a partially collapsed left upper lung lobe, a mediastinal shift, tracheal deviation to the right, and a mixed-density cystic mass containing soft tissue and fatty components located beside the bulla (Figure 2A). However, imaging features are only one of the factors that are considered in the diagnosis of this disease, and pathological examination plays a crucial role in confirming the diagnosis and understanding the specific mechanisms of the disease. Thus, we report this case to further increase the clinical understanding of this condition, focusing on an analysis of its pathological manifestations. Under thoracoscopy, wedge resection of the left upper lobe was performed. Gross examination revealed a pale red piece of tissue with a gray–white cut surface, fragile texture, and unclear structure, and focal areas showed cystic wall-like tissue. Moreover, pathological examination under low-power microscopy revealed endophytic papillary structures of varying sizes that resembled mature placental villi (Figure 2B). High-power microscopy revealed papillae lined with a single layer of cuboidal epithelial cells without atypia (Figure 2C); the papillary cores were composed of dense hyalinized stromal cells and abundant newly formed capillaries, and they exhibited varying degrees of lymphocyte and plasma cell infiltration. Focal calcification and chondroid metaplasia were observed, along with surrounding bullous changes in the adjacent lung tissue (Figure 2D). These findings help us better understand the pathology and mechanisms of this disease. Furthermore, IHC revealed that the surface cells of the lesion were positive for P-CK (Figure 2E), CK7, TTF-1 (Figure 2F), NapsinA, and EMA expression, whereas the stromal cells were positive for CD10 (Figure 2G), Vimentin (Figure 2H) and CD99 expression. Both the surface and stromal cells were negative for CEA, Desmin (Figure 2I), HMB45 (Figure 2J), S100, CD68, CD34 (Figure 2K), CD117, and Bcl-2 expression. Finally, Ki-67 staining revealed low proliferative activity in both the surface and stromal cells (approximately 1%+) (Figure 2L). The pathological diagnosis was PTL in the left upper lobe.

### 2.3 Follow-up and prognosis

Although placental transmogrification of the lung is a benign condition, both patients were followed up with regular physical examinations, chest CT scans, and basic laboratory tests to monitor for any signs of recurrence or unexpected malignant transformation. Both patients recovered well after treatment, with no evidence of recurrence or metastasis, and have maintained good overall health.

### 2.4 Patient perspective

The patient was satisfied with the diagnosis and treatment process. They felt well informed by the medical team and reported improvement in symptoms without significant side effects. The patient expressed gratitude for the thorough care and regular follow-up provided.

## 3 Discussion

PTL, which is an extremely rare, benign condition characterized by pulmonary lesions, has an estimated incidence ranging from 0.025 to 0.3%. PTL is characterized by an earlier age of onset and typically presents as a localized lesion (7). Additionally, histological examination of PTL reveals structures that resemble placental villi, although these lesions lack the biological properties of actual placental tissue. Diagnoses of PTL are made primarily based on these structural features, which, strictly speaking, do not constitute a complete definition of this disease. As a result, diagnoses that are made based on this histological pattern alone may include cases with diverse aetiologies, leading to significant heterogeneity among PTL cases. A PubMed search identified 47 relevant articles reporting a total of 59 cases that had been published as of December 2024. The clinical characteristics of these patients are summarized in Table 1, which provides an overview of their key features.

**Table 1:** Comparative features of PTL and its differential diagnoses.

Disease	Morphological features	Immunohistochemical markers	Remarks
PTL	Papillary or microcystic structures with clear stromal hyaline cells	CD10-positive stromal cells	Typically, unilateral bullous lesions; presence of clear stromal cells is characteristic
Emphysema	Unilateral bullous lesions without clear stromal cells	No specific markers	Alveolar wall destruction; absence of clear stromal cells
Hamartoma	Lacks clear stromal cells	No clear stromal cells	Relationship to PTL is unclear
Papillary invasive adenocarcinoma	Ill-defined solid nodules with invasive growth and true papillary architecture; marked epithelial atypia	No specific markers	Often accompanied by other adenocarcinoma patterns
Metastatic gestational trophoblastic disease	Predominantly in females; clinical history and HCG positivity	HCG-positive	Distinguished by clinical history and immunostaining
Sclerosing pneumocytoma	Contains solid, sclerotic, and hemorrhagic regions	TTF-1 and EMA positive	May resemble PTL morphologically
Alveolar adenoma	Well-circumscribed microcystic nodule without papillary structures	No specific markers	Clearly distinct from PTL
Pulmonary Lymphangioleiomyomatosis	Presence of HMB45-positive perivascular epithelioid cells	HMB45 positive	Immunophenotype distinct from PTL
Papillary adenoma	Simple stroma with delicate fibrovascular cores and minimal inflammation; lacks CD10-positive clear stromal cells	CD10 negative	Lacks characteristic clear stromal cells of PTL

### 3.1 Pathogenesis

Owing to the rarity of PTL and the lack of molecular biological studies on its etiology, the origin and pathogenesis of PTL are not yet fully understood, and several hypotheses have been proposed. One theory suggests that PTL could arise due to the abnormal differentiation or metaplasia of pulmonary cells or due to the benign proliferation of immature hyaline stromal cells, leading to the formation of placental-like structures that are accompanied by secondary emphysematous cystic changes. Moreover, since PTL is predominantly characterized by unilateral cystic structures and most PTL patients also have emphysema, some studies have suggested that PTL may be a variant or secondary change of emphysema, and this view that has gained widespread recognition. This view also provides a more plausible explanation for the cystic lesions that are observed in imaging studies (1, 4, 8, 9). However, emphysema is a diffuse disease, and patients with emphysema are often have deficient in alpha-1 antitrypsin. In contrast, PTL (localized pulmonary lesion) is typically a localized condition, and to date, there have been no reports of alpha-1 antitrypsin deficiency in PTL patients (10). Another study proposed that PTL arises from lipomatosis due to the presence of fat tissues inside the villi (11). Another hypothesis posits that PTL may arise as a reactive or compensatory process secondary to chronic lung injury or inflammation, for example, in patients with a history of smoking, spontaneous pneumothorax, or bullous lung disease. In the context of chronic lung injury or inflammation, dysregulated growth of the respiratory epithelium, driven by cytokines that are produced by mast cells, promotes the proliferation of respiratory epithelial cells, resulting in the coverage of hamartomatous components by hyperplastic respiratory epithelium (12). Additionally, congenital malformations or secondary lesions that are caused by hyperplasia of lymphatic or vascular structures within the lung parenchyma may also contribute to PTL development (11, 13).

**Figure 3:**
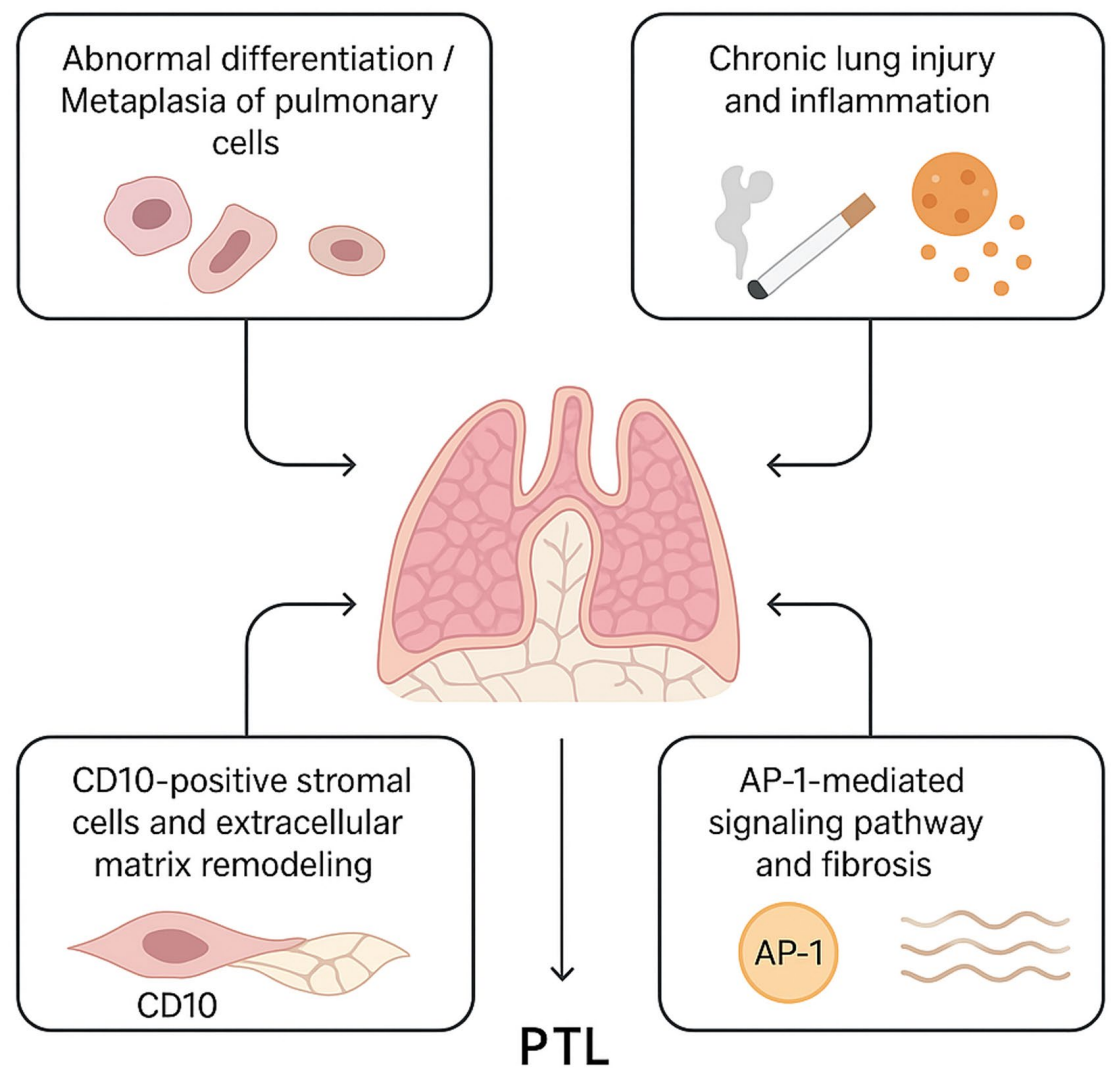
Proposed Pathogenesis Model of PTL. (1) Aberrant differentiation or metaplasia of respiratory epithelial and immature stromal cells leads to placental-like papillary structures. (2) Chronic lung injury or inflammation, including smoking or pneumothorax history, triggers mast cell-mediated cytokine release, promoting epithelial hyperplasia. (3) CD10-positive stromal cells participate in extracellular matrix remodeling and interact with epithelial cells. (4) AP-1-mediated signaling regulates cellular proliferation and fibrosis, contributing to cystic changes and tissue remodeling characteristic of PTL.

In addition, a recent study revealed that JUND, COL4A2, COL6A2, IGFBP5, and IGFBP7 are consistently upregulated in PTL (localized pulmonary lesion), supporting the hypothesis that AP-1-mediated signaling may contribute to fibroproliferative changes within the lesion (14). These molecular alterations could be linked to the activation of CD10-positive stromal cells, which in turn might promote extracellular matrix remodeling and cystic transformation. Together with other proposed mechanisms—such as abnormal differentiation or metaplasia of pulmonary cells, lipomatosis, and reactive processes—these findings suggest that multiple, possibly interconnected pathways may underlie PTL development (Figure 3). However, owing to the rarity of this condition and the absence of large-scale molecular studies, the precise pathogenic sequence remains unclear. Further research integrating histopathological, immunophenotypic, and genomic data is warranted to clarify the molecular and cellular events driving PTL.

### 3.2 Clinical characteristics

According to reports in the literature of more than 59 cases, most patients with PTL (localized pulmonary lesion) do not exhibit specific clinical manifestations. Some patients may present with symptoms such as dyspnoea, chest pain, and cough. Thus, PTL is often discovered incidentally due to accompanying conditions, including pleural effusion, chronic obstructive pulmonary disease (COPD), pneumothorax, lung adenocarcinoma, Swyer–James (MacLeod) syndrome, and ductal carcinoma *in situ* of the breast (7, 15–18). Currently, only two cases of PTL (localized pulmonary lesion) associated with cancer have been reported in the literature (5, 16). Therefore, there is a lack of case report data suggesting a relationship between PTL and cancer. The age of onset of patients ranges from 14 to 72 years (with an average age of 42 years), and PTL predominantly affects middle-aged males. Most cases present as cystic lesions, whereas a few appear as solitary solid pulmonary nodules. However, the nodules are not always entirely solid and often contain several small, round cavities (19). Moreover, there are not enough data available to show predilection of laterality or lobe.

This patient in case 2, the presence of both a long-term smoking history and a prior pulmonary bulla raises the question of whether smoking may be a potential risk factor for PTL or merely a coincidental finding. Although the exact pathophysiological link between smoking, bullous emphysema, and PTL remains speculative, chronic cigarette exposure has been associated with structural lung changes and inflammatory responses that could predispose to abnormal mesenchymal proliferation. Additionally, approximately 41% of patients with PTL described in the literature had a history of smoking, suggesting that smoking may be a factor that potentially induces PTL development (20). Further studies are needed to clarify whether smoking plays a causal role in PTL development or simply coexists as a common background factor in affected individuals.

### 3.3 Radiological appearance of PTL

The radiological appearance of PTL depends mainly upon the clinical presentation and whether PTL is associated with bullous emphysema or hamartoma. Studies have shown that the radiological appearance of PTL can be categorized into three distinct patterns: (I) bullous emphysema, which is the most common pattern; (II) a mixed pattern of cystic lesions and nodules; and (III) the rare solitary nodule pattern (18). When a solitary nodule is identified, the radiographical appearance may or may not be typical of a hamartoma (21). Positron-emission tomography demonstrated an SUVmax of 9.3 (22). Additionally, PTL can present with the typical clinical and radiographical appearance of tension pneumothorax owing to the rupture of bullae.

### 3.4 Pathological characteristics

Gross samples of PTL often exhibit a well-defined cystic-solid structures. On the cut surface, clusters of vesicles, villi, or granular tissue can be observed, resembling the immature grape-like structure of placental tissue. No definite pleural indentation is observed, but collapse of normal lung parenchyma may be observed. Under the microscope, PTL lesions consist of multiple papillary structures that resemble immature placental villi. The papillary surfaces are covered with cuboidal epithelial cells without atypia. The papillary cores, or stromal components, contain dense hyaline cells and abundant newly formed capillaries. In some cases, mature adipose tissue, smooth muscle tissue, or even calcification can be observed, along with varying degrees of lymphocytic infiltration (3, 23, 24). The nuclei of the clear stromal cells are round or oval, with fine chromatin, and exhibit no atypia or mitotic activity. When stromal oedema or hyaline degeneration occurs, the number of stromal cells is reduced. Additionally, the lesions are often accompanied by surrounding pulmonary bullae or hamartomatous components (25). In focal areas, airway structures are preserved, which is the only normal anatomic structure available according to which the organ can be recognized. However, a high degree of clinical suspicion is required for diagnosis.

### 3.5 Immunohistochemical characteristics

The cuboidal epithelial cells covering the surface of the papillae in PTL lesions were diffusely positive for pan-cytokeratin (P-CK), CK7, TTF-1, Napsin A, and EMA, indicating their epithelial origin. In contrast to some previous reports of focal expression of TTF-1, Napsin A, and EMA, in our cases these markers showed diffuse positivity. The stromal cells in the papillary cores were positive for Vimentin and CD10 in both cases, with case 1 showing focal CD117 positivity and case 2 showing CD99 positivity. Both surface and stromal cells were negative for markers including CEA, Desmin, S100, HMB45, CD68, CD34, SMA, and HCG. Notably, CD10 positivity in stromal cells was consistently observed, suggesting a possible role in the pathological process of PTL. Ki-67 staining revealed low proliferative activity in both cell populations (approximately <5% in case 1 and around 1% in case 2), supporting the benign nature of the lesions. These findings provide further insight into the biphasic epithelial and mesenchymal differentiation of PTL and aid in distinguishing it from other cystic or papillary lung lesions.

### 3.6 Differential diagnosis

PTL should be differentiated from other pulmonary lesions that exhibit papillary or microcystic structures with a cuboidal epithelial lining and thus morphologically resemble PTL. These include emphysema, hamartoma, papillary invasive adenocarcinoma, metastatic gestational trophoblastic disease, sclerosing pneumocytoma, alveolar adenoma, pulmonary lymphangioleiomyomatosis, and papillary adenoma. First, studies have demonstrated that unilateral bullous lesions are an important differential in patients with PTL, especially under circumstances in which emphysema is unlikely (2); however, emphysema lesions do not contain clear stromal cells. Hamartomas lack clear stromal cells, and the relationship between PTL and hamartomas remains unclear (11). Papillary invasive adenocarcinoma typically presents as ill-defined solid nodules with invasive growth, a true papillary architecture, and significant epithelial atypia, and it is often accompanied by other adenocarcinoma patterns. Metastatic hydatidiform moles, which are predominantly observed in females, can be distinguished on the basis of clinical history and immunohistochemical staining for HCG. Sclerosing pneumocytoma may resemble PTL but includes solid, sclerotic, and haemorrhagic regions, with stromal cells that are positive for TTF-1 and EMA expression. Alveolar adenoma appears as a well-defined microcystic nodule but lacks papillary structures. Pulmonary lymphangioleiomyomatosis features HMB45-positive perivascular epithelioid cells. Papillary adenoma has a simple stroma with delicate fibrovascular cores and minimal inflammation, lacking CD10-positive hyaline stromal cells. A comparative summary of key morphological and immunohistochemical features of PTL and its differential diagnoses is presented in Table 1. Thus, a definitive diagnosis requires the integration of clinical information, imaging findings, histopathological morphology, and immunohistochemical analysis.

### 3.7 Treatment and prognosis

PLT is considered to be a benign lesion condition, and there is only one reported case of transformation into papillary adenocarcinoma in the literature. Therefore, timely diagnosis is particularly important. Conservative treatment has shown no significant efficacy, and if a patient has coexisting pulmonary bullae, the progression of bullae may impair normal lung function (26). Currently, surgical resection is the preferred treatment method. The goal is to minimize the extent of surgery and preserve as much normal lung tissue as possible (27). There is no evidence to support the routine use of chemotherapy or radiotherapy. The overall prognosis of PTL appears favorable, even among patients presenting with severe pulmonary symptoms. To date, no cases of recurrence or malignant transformation after complete surgical excision have been documented. However, given the limited number of reported cases and the scarcity of long-term follow-up data, this conclusion should be interpreted with caution. Long-term surveillance is therefore recommended to better understand the biological behavior and potential risks associated with PTL.

## 4 Conclusion

This study reports two cases of PTL, including the results of comprehensive immunohistochemical analysis, and provides a review and summary of the relevant literature. These lesions exhibit diverse morphologies under microscopy but consistently have placental-like structures and CD10-positive clear stromal cells. However, owing to the limited research on PTL, whether PTL is a neoplastic lesion as well as the nature and pathogenesis of clear stromal cells within the papillary stroma remains unclear.

CD10 is a zinc-dependent metalloproteinase with elastase activity capable of degrading elastic fibers within lung tissue (28, 29), consistent with the pathological degeneration of elastic fibers observed in the alveolar septa. In the two cases reported here, CD10 expression was detected in stromal hyaline cells, aligning with findings from most previous studies. CD10-positive cells exhibit plasticity, transitioning among lipoblasts, mature adipocytes, and fibroblasts. Some extracellular matrix components also stain positive for CD10, and these areas frequently show abundant elastic fiber deposition, suggesting that CD10-positive stromal cells not only possess multidirectional differentiation potential but may also actively participate in the synthesis and metabolism of elastic fibers and the extracellular matrix.

Given CD10’s elastase function, it may contribute to PTL pathogenesis by regulating the degradation and remodeling of elastic fibers, thereby promoting cystic degeneration and stromal proliferation within lung tissue. Moreover, Cavazza et al. used microdissection to isolate clear stromal cells from PTL lesions, revealing significant microsatellite instability distinct from surrounding lymphocytes, indicating clonality of these cells (15). This supports the notion that PTL is a benign mesenchymal tumor arising from clonal proliferation of immature stromal cells rather than a subtype of emphysema. Furthermore, CD10-positive cells may mediate local extracellular matrix degradation and remodeling through enzymatic activity, facilitating fibrosis and advancing cystic changes. The clonal proliferation of these stromal cells suggests tumor-like behavior, distinct from reactive or inflammatory processes. Such proliferation may not be limited to stromal compartments but could also influence adjacent alveolar epithelial cells, contributing to pathological remodeling. At the molecular level, studies indicate that the AP-1 signaling pathway may regulate CD10 expression and stromal cell proliferation. Activation of AP-1 can promote fibroblast-like cell proliferation and extracellular matrix production, enhancing local fibrotic responses and furthering PTL lesion progression. This mechanism provides a potential signaling explanation for the role of CD10-positive stromal cells in PTL pathogenesis. Additionally, immunohistochemical analysis shows that the epithelium lining papillary structures is positive for cytokeratin and TTF-1 but negative for calretinin, WT1, and podoplanin, suggesting a respiratory epithelial origin rather than pleural. This implies that differential CD10 expression within the respiratory epithelium may be a critical factor in PTL development, with epithelial-stromal interactions potentially driving lesion formation.

In summary, PTL is considered to be a rare benign pulmonary lesion. CD10-positive stromal cells likely play a pivotal role in PTL cystic degeneration and stromal proliferation through their elastase activity, multidirectional differentiation potential, and clonal expansion. Further elucidation of the regulatory mechanisms controlling CD10 expression, including its relationship with AP-1 signaling, may clarify PTL pathogenesis and offer novel diagnostic and therapeutic targets. Because PTL can lead to complications such as pneumothorax and pleural effusion, timely diagnosis and treatment of this condition are particularly important. Surgical resection offers both diagnostic and therapeutic advantages for PTL patients, whereas conservative treatment may result in disease progression and impaired lung function.

## Data Availability

The original contributions presented in the study are included in the article/Supplementary material, further inquiries can be directed to the corresponding author.
